# Phase noise reveals early category-specific modulation of the event-related potentials

**DOI:** 10.3389/fpsyg.2014.00367

**Published:** 2014-04-24

**Authors:** Kornél Németh, Petra Kovács, Pál Vakli, Gyula Kovács, Márta Zimmer

**Affiliations:** ^1^Department of Cognitive Science, Budapest University of Technology and EconomicsBudapest, Hungary; ^2^DFG Research Unit Person Perception, Friedrich Schiller University of JenaJena, Germany; ^3^Institute of Psychology, Friedrich Schiller University of JenaJena, Germany

**Keywords:** phase noise, category effect, P1, N170, P2

## Abstract

Previous studies have found that the amplitude of the early event-related potential (ERP) components evoked by faces, such as N170 and P2, changes systematically as a function of noise added to the stimuli. This change has been linked to an increased perceptual processing demand and to enhanced difficulty in perceptual decision making about faces. However, to date it has not yet been tested whether noise manipulation affects the neural correlates of decisions about face and non-face stimuli similarly. To this end, we measured the ERPs for faces and cars at three different phase noise levels. Subjects performed the same two-alternative age-discrimination task on stimuli chosen from young–old morphing continua that were created from faces as well as cars and were calibrated to lead to similar performances at each noise-level. Adding phase noise to the stimuli reduced performance and enhanced response latency for the two categories to the same extent. Parallel to that, phase noise reduced the amplitude and prolonged the latency of the face-specific N170 component. The amplitude of the P1 showed category-specific noise dependence: it was enhanced over the right hemisphere for cars and over the left hemisphere for faces as a result of adding phase noise to the stimuli, but remained stable across noise levels for cars over the left and for faces over the right hemisphere. Moreover, noise modulation altered the category-selectivity of the N170, while the P2 ERP component, typically associated with task decision difficulty, was larger for the more noisy stimuli regardless of stimulus category. Our results suggest that the category-specificity of noise-induced modulations of ERP responses starts at around 100 ms post-stimulus.

## INTRODUCTION

There has been a long tradition of applying external noise to visual stimuli in the last two decades of the 20th century in visual psychophysics as well as in studies of face perception to study various stages of visual processing (Costen et al., 1994; Gold et al., 1999; Näsänen, 1999). Common methods included noise manipulation combined with electrophysiological and brain imaging methods to investigate and identify the underlying neuronal mechanisms of the various functions of the perceptual system. In recent studies, different types of external noise were used, including uniform white noise (Wild and Busey, 2004), Gaussian noise (Jemel et al., 2003), bit noise (Smith et al., 2012), multiplicative noise combined with brain imaging techniques (e.g., Schyns et al., 2003, 2007, 2009; Smith et al., 2004, 2006, 2007, 2008, 2009; Rutishauser et al., 2011), Fourier phase-randomization techniques (Rousselet et al., 2008a; Bankó et al., 2011) with the mean-phase randomization (Dakin et al., 2002), and pink noise (Tjan et al., 2006; Rousselet et al., 2008a,b). These techniques provided valuable insights into the spatial and temporal events at different cortical regions in the human brain involved in different stages of face processing.

Regarding human face perception, electrophysiological studies have described a large positive (P1) and negative (N170) wave over the occipital and posterior occipito-temporal areas that might be sensitive to face stimulation (Bentin et al., 1996; Eimer, 2000a; Itier and Taylor, 2004). As of today, usually the N170 is considered as the first clearly face-sensitive event-related potential (ERP) component, although category-specific processes have been suggested by some studies to be present already at 100 ms (or even 50-80 ms) after stimulus onset (corresponding to the P1 component; George et al., 1997; Seeck et al., 1997; Liu et al., 2002; Herrmann et al., 2005a; Thierry et al., 2007). The N170 is higher in amplitude and shorter in latency to pictures of faces than to exemplars of other non-face object categories (Bentin et al., 1996; for reviews see Rossion and Jacques, 2008, 2011; Eimer, 2011). Recently, however, the specificity of N170 for faces has been questioned by studies that failed to demonstrate higher N170 amplitude for faces when compared with cars (Rossion et al., 2000a; Schweinberger et al., 2004; Thierry et al., 2007; Dering et al., 2011; Kloth et al., 2013).

With regard to noisy stimulation, Jemel et al. (2003) used a parametric design to characterize early ERPs to face stimuli embedded in gradually decreasing levels of random Gaussian noise. The authors found that while the P1 component was unaffected by noise levels, there was a linear increase in the amplitude and a decrease in the latency of the N170 with decreasing levels of noise. Jemel et al. (2003) concluded that while the early P1 component is likely to reflect the stage at which the perceptual analysis of faces is achieved, the N170 seems to reflect the successful categorization of faces (Liu et al., 2002; Jemel et al., 2003). In other words, earlier ERP components might reflect the extraction of task-relevant information from noisy stimuli. This modulation of the N170 component is in line with findings showing attenuated and delayed N170 to faces either without internal features or in the absence of their contours (Eimer, 2000a). In addition, Rousselet et al. (2008b) found that sensitivity to phase noise falls in the time window of the N170 (130–170 ms).

The P2 ERP component is characterized by a positive-going deflection over lateral occipito-temporal areas and a maximal peak between 200 and 250 ms. Recently, it has been shown that the amplitude of the P2 is sensitive to the inversion of either the entire face or of its parts (Milivojevic et al., 2003; Boutsen et al., 2006) and has been linked to the processing of spatial relations between facial features in individual faces (Latinus and Taylor, 2006). Wiese et al. (2009) have shown that own race faces generate larger P2 components when compared with faces of other races, although, this effect interacts with expertise (Stahl et al., 2008). Larger P2 was also reported for younger when compared to older face stimuli (Stahl et al., 2008). Furthermore, it has been suggested that the P2 is involved in individual face recognition mechanisms (Halit et al., 2000). Altogether, these results suggest that the P2 is involved in the deeper and more advanced analysis of faces when compared to earlier components. Regarding noisy stimulation, Rousselet et al. (2007, 2008a) showed that the P2 is larger to noise patterns in comparison to faces. In a follow-up study, they tested whether this difference was independent from the changes of the N170 amplitude and therefore a peak-to-peak analysis was carried out on the modeled data (Rousselet et al., 2008b). The authors found that the P2 difference is a simple carry-over effect that was present already on the N170. In addition, the P2 was identified as a clear neural correlate of decision difficulty under noisy stimulation (Philiastides et al., 2006; Heekeren et al., 2008). However, a recent study using image warping as well as phase noise to manipulate task difficulty found that rather, the P2 reflects noise-sensitive increases of sensory processing and not task difficulty *per se* (Bankó et al., 2011). In a previous ERP study, we confirmed these results and distinguished the nature of adding phase noise from that of another irrelevant, overlapping car image (Nagy et al., 2009). We found that adding phase noise reduces the N170 component, while the amplitude of the P2 component increases with the amount of noise added. In addition, the P2 was larger in the phase noise condition than if another coherent, but irrelevant stimulus (a car) was added to the face.

In general, adding noise to face images leads to smaller N170 amplitudes, reflecting impaired early structural face processing (Bentin and Deouell, 2000; Eimer, 2000a,b for a review see Rossion and Jacques, 2008), as well as to larger P2 amplitudes. However, the effect of noise reflected in the early P1 component is equivocal as of today. While Jemel et al. (2003) found that the effect of added noise does not affect P1 amplitude, other studies have demonstrated that the P1 and P2 components are significantly larger in the noise-present when compared with noise-absent conditions (e.g., Curran et al., 1993; Tucker et al., 1994; Mercure et al., 2008; Bankó et al., 2011).

To the best of our knowledge, so far no study has explicitly compared the noise-dependence of face and non-face stimulus categories. The goal of the present study was to test whether adding phase noise to stimuli affects the neural processing of different high-level categories, such as faces and cars, in a similar way.

## MATERIALS AND METHODS

### PARTICIPANTS

Sixteen naïve, healthy volunteers (two left-handed, eight females, mean age: 22.1 years ± 2.1 years SD) participated in the study. They received partial course credits for their participation and gave signed, informed consent in accordance with the Ethical Committee of the Budapest University of Technology and Economics prior to testing. All participants had normal or corrected-to-normal visual acuity, no previous history of any neurological or ophthalmologic diseases and were not under medication. Three participants were excluded from the final electrophysiological analyses due to insufficient numbers of ERP segments after artifact rejection. Therefore, statistical analysis was conducted on the data of thirteen subjects (seven females, one left-handed, mean age: 21.5 years ± 1.8 years SD).

### STIMULI

**FIGURE 1:**
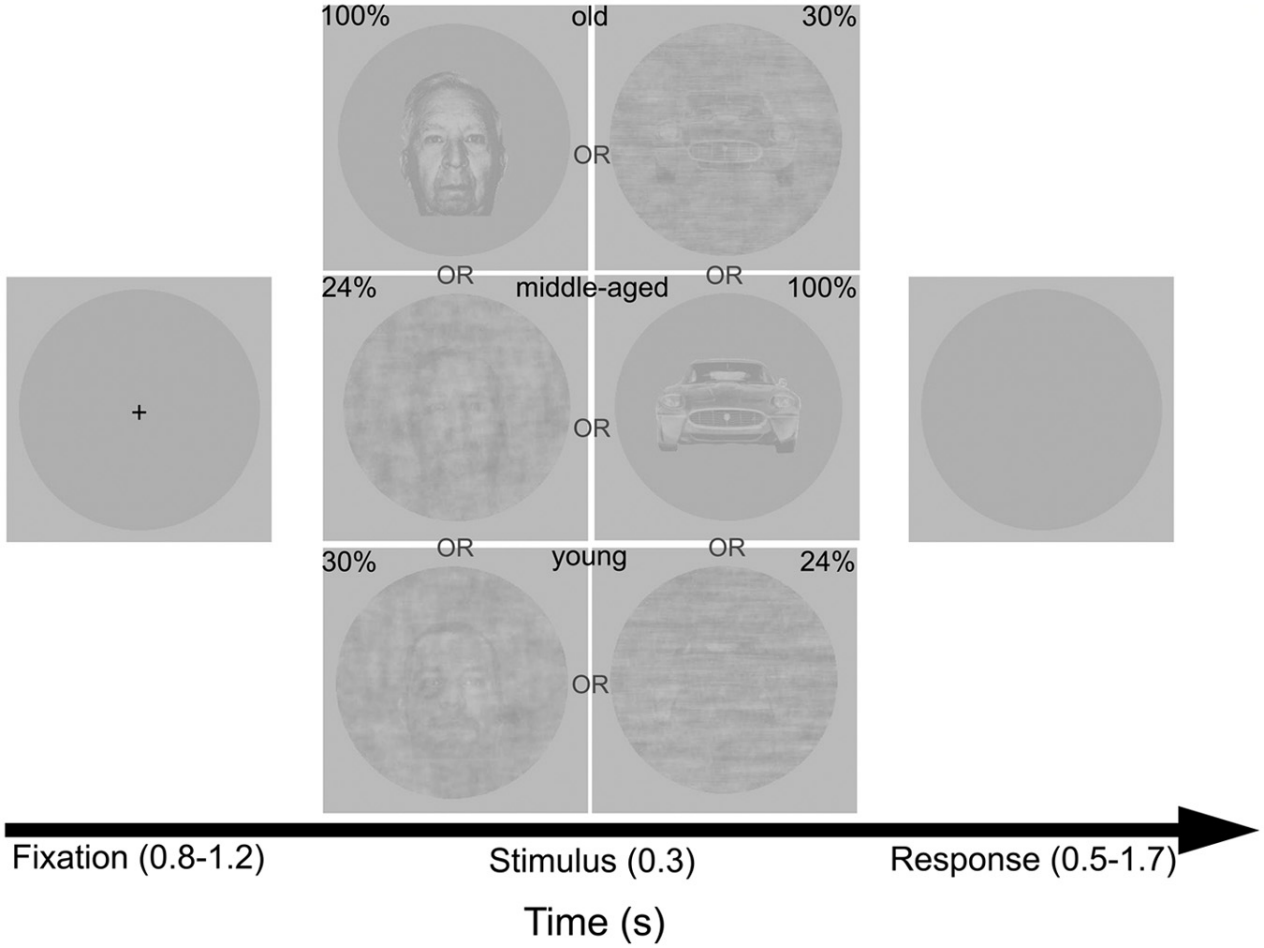
**Procedure and sample stimuli.** Timeline depicts some examples of faces and car test stimuli at different levels of phase coherences (100, 30, or 24%) and with different ages (young, middle-aged, or old).

Front-view grayscale images of faces and cars were used with age gradually changing, with or without phase noise. Face stimuli were digital images of six Caucasian males from a larger face database (Minear and Park, 2004). Three of them were younger than 30 years old, while the others were older than 60 years old. Car images were old and new variations of the same models of three well-known commercial car types (VW, Mercedes, and Jaguar), and were downloaded from freely available websites. Car images were presented in full frontal views, similar to those of Kloth et al. (2013). All images were first converted into grayscale (8 bit) using Adobe Photoshop CS3 Extended 10.0 (Adobe Systems Inc.). Stimuli of both categories were then revealed through a circular aperture (radius = 153 pixels). Stimulus size was equated for each category (mean height and width of the faces and cars were 248 and 154 pixels, and 153 and 251 pixels, respectively; see **Figure 1**). Since previous studies have shown that early ERP components, such as P1, are sensitive to luminance (Johannes et al., 1995) and that neural processes are sensitive to luminance histogram skewness (Olman et al., 2008), we have equated all stimuli in luminance and matched their histograms using the *lummatch* and *histmatch* functions of the SHINE toolbox (Willenbockel et al., 2010). On the other hand, we did not equate the spectral content of the images, as we would concurrently have manipulated artificially the difficulty of the age-discrimination task for the face stimuli. It is well known that facial aging is reflected in the dynamic, cumulative effects of the skin, and is a complex synergy of skin textural changes and the loss of facial volume (Coleman and Grover, 2006). The decreased tissue elasticity and the redistribution of subcutaneous fullness result in a larger amount of higher spatial frequency information. This low-level difference between younger/newer and older individuals does not appear when comparing new cars to old ones.

In order to increase task difficulty, two different types of stimulus manipulations were applied. First, we decreased the age difference between young and old stimuli using a warping algorithm (Winmorph 3.01; Kovács et al., 2005, 2006, 2007; Bankó et al., 2011). That is, we paired a young and an old image of the same category and created a morph continuum with seven intermediate images of faces and cars. Second, the coherence of the original images (100% phase coherence) and the intermediate morphs was manipulated by decreasing their phase coherence in two steps (30 and 24% phase coherences, respectively) using the weighted mean phase technique (Dakin et al., 2002). In fact it means that we have manipulated the phase coherence of the RGB values (and not the luminance values) of the stimuli. This phase-randomization resulted in the gradual elimination of the cues important for accurate age judgments.

To avoid behavioral ceiling or floor-effects and to have comparable performance for face and car stimuli, first we performed a behavioral pilot experiment (*n* = 12). We tested the age discrimination performance of participants for 10 exemplars of faces and cars as well as for 10 incrementally graded noise levels from 0 to 100% phase coherence. For the final three stimulus-pairs, morph levels of the young–old continuum and the exact percentage of phase noise were selected based on the results of this pilot study, so that the average age-discrimination performance would be similar across faces and cars for each phase noise level.

Stimuli were presented centrally on a uniform gray background on a 26 inch LCD monitor at a refresh rate of 60 Hz, while viewing distance (57 cm) was maintained using a chinrest. Stimulus presentation was controlled by MATLAB 2008a (Mathworks, Natick, MA, USA) using Psychtoolbox 3.0.9 (Brainard, 1997; Pelli, 1997) and custom-made scripts.

### PROCEDURE

As it is generally more difficult to determine the age of a car than the age of a face, as suggested by the results of the pilot study, first, participants were presented with a practice session for the car stimuli prior to the experiment.

#### Practice experiment

In the first part of the practice, participants had to choose the younger (newer) car from a pair of stimuli, depicting the endpoints of the morph continuum, or in other words the oldest and youngest versions of a model. Each pair was presented eight times (exposition time = until response; inter-trial interval = 500 ms). The newer model was displayed randomly on either the left or the right side. Participants received feedback after each trial as well as at the end of the block. Participants performed at least four, but not more than six blocks of 24 trials. The practice was interrupted if 90% correct performance was reached in two consecutive blocks. A subject was excluded from the study if their performance did not reach this criterion even after 10 practice blocks (0 participants).

Second, participants performed an age-discrimination task on individually presented cars depicting the endpoints of morph continuums. In this part of the practice a fixation screen was presented in the beginning of each trial for a random time between 800 and 1200 ms, followed by the presentation of the test image (100% phase coherence) for 300 ms. Participants were instructed to respond within 2 s after stimulus onset (inter-trial interval = 800 ms). Within a single block, car stimuli were presented in a random order. Subjects had to perform 4–6 blocks of 24 trials (each car presented four times in a random order). The practice was interrupted if 90% correct performance was reached in two consecutive blocks. A subject was excluded from the study if her/his performance did not reach this criterion even after six practice blocks (0 participants).

Finally, immediately prior to the ERP recording experiment, participants were asked to passively fixate the center of each stimulus (both faces and cars) at each noise level and at each morph level for 5000 ms (inter-stimulus interval 1000 ms) for the subjects once, to avoid strong familiarity effects of the practice phase with cars.

#### ERP recording experiment

Subjects performed an old vs. young age discrimination task for faces and cars. The trial structure was identical to that of the second task of the practice experiment (**Figure 1**). Noise-levels, stimulus categories, and morph levels were intermixed and presented in random order within each block. Each participant completed eight blocks of 378 trials [2(category; face vs. car) × 3(exemplars; face morph-continuum vs. car morph-continuum) × 3(coherence level, 100% vs. 30% vs. 24%) × 7(morph level) × 3(number of repetitions)]. Subjects were allowed to take a short break between blocks. An experimental session lasted approximately 100 min.

### BEHAVIORAL DATA ANALYSIS

Accuracy and response times (RTs) were collected during the experiment. Performance was assessed by computing just noticeable differences (JND) as the smallest difference in morph level required to perform the old versus young age discrimination task reliably (Lee and Harris, 1996; Bankó et al., 2009) for each stimulus type individually. First, psychophysical data were modeled by the cumulative Gaussian psychometric function, using the *Psignifit* toolbox (Version 2.5.6.) for MATLAB (Wichmann and Hill, 2001). JNDs were calculated using the equation JND = (Perf_75_–Perf_25_)/2, where Perf_75_ and Perf_25_ denote the morph levels leading to 75 and 25% accuracies, respectively. JNDs for different stimuli and noise levels were calculated separately. RTs were calculated as the average of the RTs for stimuli yielding 25 and 75% performance. JNDs and RTs were analyzed with a 2 × 3 repeated measures ANOVA with category (2; face vs. car) and phase coherence (100% vs. 30% vs. 24%) as within-subject factors. *Post hoc t-*tests were computed using Fisher’s Least Significant Difference (LSD) tests.

### ELECTROPHYSIOLOGICAL RECORDING AND ANALYSIS

#### EEG acquisition and processing

Electroencephalography (EEG) data was recorded using a Brain-Amp (BrainProducts GmbH, Munich, Germany) amplifier from 60 Ag/AgCl scalp electrodes placed according to the international 10/10 electrode system (Chatrian et al., 1985) and mounted on an ActiCap (Easycap, HerrschingBreitbrunn, Germany). Additionally, four periocular electrodes were placed at the outer canthi of the eyes and above and below the right eye for recording the electrooculogram (EOG). All channels were referenced to FCz online and digitally transformed to a common averaged reference offline. The ground was placed at AFz and all input impedances were kept below 10 kΩ. EEG was digitized at a 1000 Hz sampling rate with an analog bandpass filter of 0.016–1000 Hz. Subsequently, a digital 0.1 Hz, 12 dB/octave Butterworth zero phase high-pass filter was used to remove DC shifts, and a 50 Hz notch filter was applied to minimize line-noise artifacts. Finally, a 12 dB/octave low-pass filter with a cut-off frequency of 50 Hz was applied. Trials that contained voltage fluctuations exceeding ±100 μV, or eye blinks exceeding ±50 μV were rejected.

#### ERP data analysis

After the eye blink artifacts were corrected (Gratton et al., 1983) the EEG was segmented offline using Brain Vision Analyzer 1.05.0002 (Brain Products GmbH, Munich, Germany) into 1300 ms epochs using a 500 ms pre stimulus interval. Segments were baseline corrected over the 500 ms prestimulus window, artifact rejected, and averaged to obtain the ERP waveforms for each subject and for each condition. Individual ERPs were averaged to compute the grand average ERP for visualization. Statistical analysis was performed on the early visual components P1, N170, and P2 of the individual average ERP waveform. The peak amplitude and latency of the individually averaged ERPs was extracted using a semiautomatic detection algorithm that identified the global maxima separately for each selected channels in a specific time window. P1 was defined as a main positive deflection in the 80–130 ms time window. N170 was defined as a negative component at around 130–200 ms after stimulus onset, and P2 as a second positive component in the 200–250 time window. P1 amplitude was measured over O1, PO7 (left hemisphere, LH), and O2, PO8 (right hemisphere, RH) electrode positions. In the case of the N170, the usual posterior-occipito-temporal sites, corresponding to the PO7, PO9, P7, and P9 (LH) and PO8, PO10, P8, and P10 (RH) were used, while P2 amplitude was measured over PO3, PO7, O1 (LH), and PO4, PO8, and O2 (RH) channels. Both amplitude and latency values of the pooled values of the relevant electrodes were entered into a four-way repeated-measures ANOVA with hemisphere (2; left vs. right), category (2; face vs. car), coherence (3; 100% vs. 30% vs. 24% phase coherence), and age (3; young/new vs. middle-aged vs. old) as within-subject factors separately for each component. The Greenhouse–Geisser correction was applied to correct for possible violations of sphericity. *Post hoc* tests were computed using Fisher’s LSD tests.

## RESULTS

### BEHAVIORAL RESULTS

**FIGURE 2:**
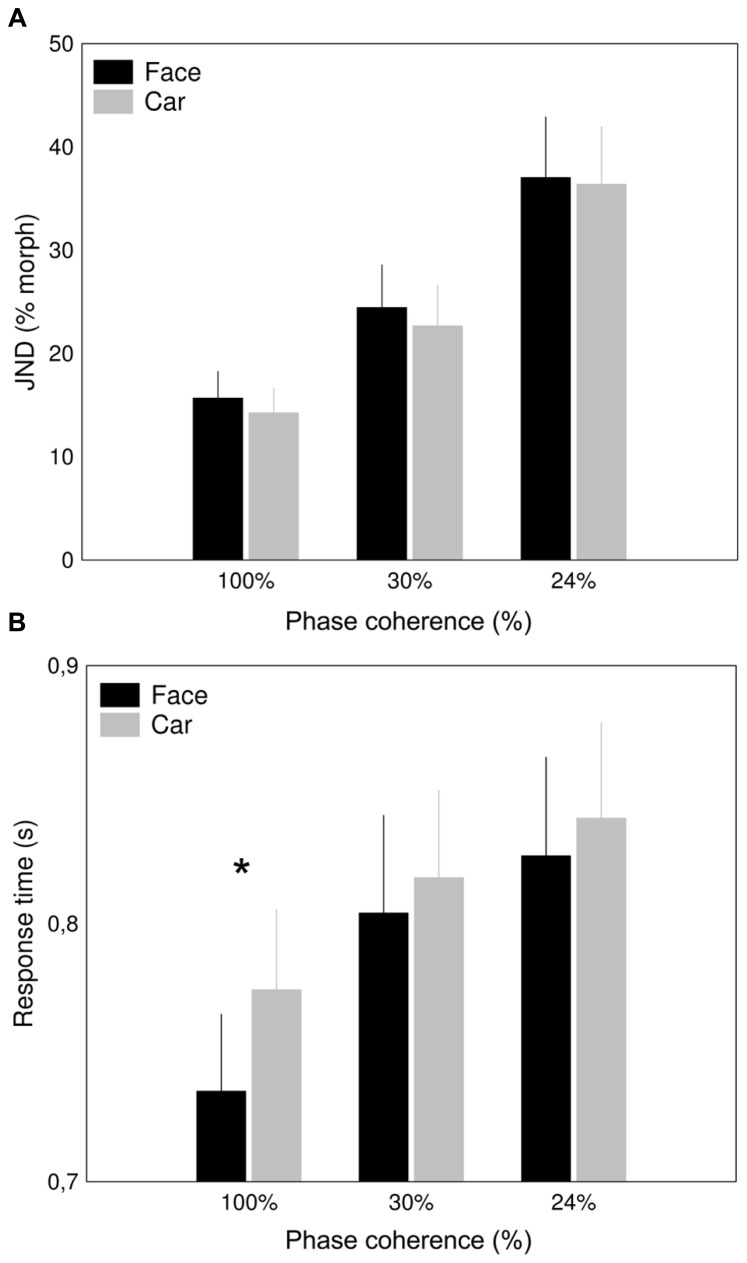
**Behavioral results.** Effect of added noise on the accuracy **(A)** and response times **(B)** in the age discrimination task for faces (black columns) and for cars (gray columns). Just noticeable differences (JND; ±SD) were calculated (see Materials and Methods) to characterize the performance of the subjects. The *x*-axis denotes different levels of phase coherences (*p < 0.05).

The age-discrimination performance of the participants was similar for faces and cars (main effect of category: *F*(1,15) = 0.198, *p* = 0.661, η^2^ = 0.013; **Figure 2A**), suggesting that the difficulty of the task was similar for the two stimulus categories. As expected, additional phase noise reduced the performance incrementally (main effect of coherence: *F*(1.11,16.58) = 13.002, *p* < 0.0001, η^2 ^= 0.464). This effect was similar for the two stimulus categories, as suggested by the lack of interaction between category and coherence (*F*(2,30) = 0.0461, *p* = 0.955, η^2 ^= 0.003).

Paralleling performance results, RTs were also prolonged by reduced phase-coherence (main effect of coherence: *F*(1.1,16.54) = 23.98, *p* < 0.0001, η^2 ^= 0.62, **Figure 2B**). In addition, significantly longer RTs were found for car stimuli when compared to faces [main effect of category: *F*(1,15) = 5.47, *p* = 0.03, η^2 ^= 0.27], at least for the 100% coherence condition (category × coherence level interaction: [*F*(2, 30) = 3.316, *p* < 0.05, η^2 ^= 0.181].

### RESULTS OF THE ELECTROPHYSIOLOGICAL MEASUREMENT

**FIGURE 3:**
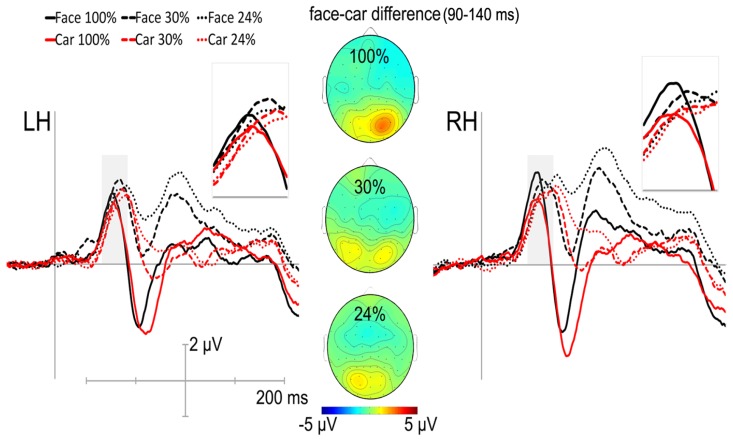
**Grand average ERPs displayed between -100 and 500 ms of the pooled posterior-occipito-temporal recording sites of the N170 for the left (LH) and for the right hemisphere (RH).** 100% phase coherence: thick line, 30% phase coherence: dashed line, 24% phase coherence: dotted line; for faces (black) and cars (red), respectively. Insets depict the category-specificity of noise-induced modulations on the P1 component. Topographical voltage maps of ERP differences between faces and cars at different phase coherence levels (100%-upper, 30%-middle, 24%-lower) show hemispheric asymmetries in the P1 time window. Positivity is red.

The stimuli evoked ERPs with clearly identifiable P1, N170, and P2 components, measured at occipital and posterior-occipito-temporal sites. **Figure 3** depicts the grand average ERPs of the pooled recording sites over the LH and RH, displayed between -100 and 500 ms.

#### P1

**FIGURE 4:**
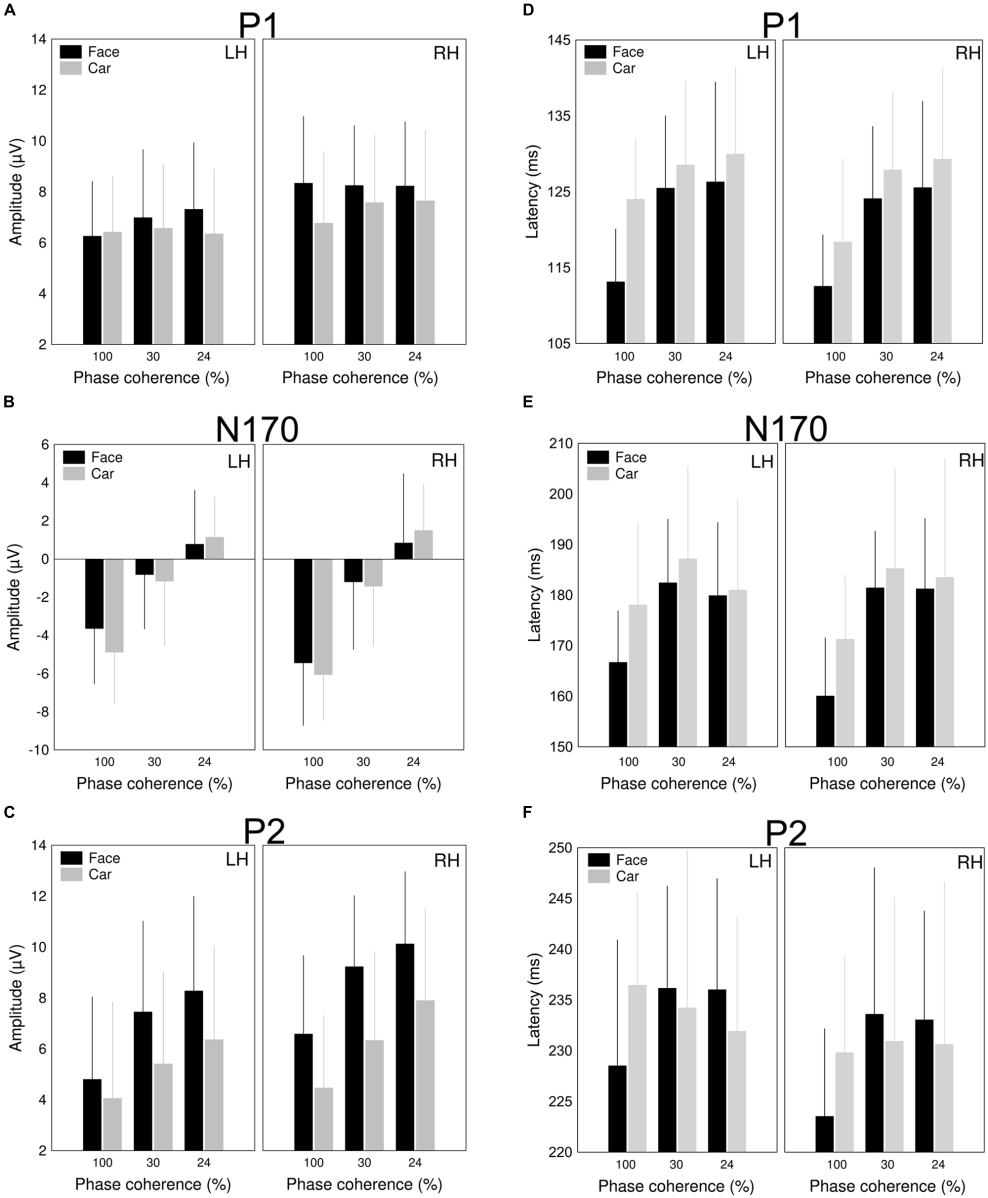
**Mean (±SD) of the amplitudes and latencies of the (A,D) P1, (B,E) N170, and (C,F) P2 components for faces (black columns) and cars (gray columns) at different levels of phase coherences**.

Significantly larger P1 amplitudes were observed for faces when compared to car stimuli [main effect of category: *F*(1,12) = 10.16, *p* = 0.008, η^2 ^= 0.46]. Importantly, the noise-induced modulation of the P1 component showed category-specificity in a hemisphere-specific manner [hemisphere × category × coherence interaction: *F*(2,24) = 8.8452, *p* < 0.01, η^2 ^= 0.4243], as it was enhanced as a result of adding noise to the images over the RH for cars (*post hoc* test for 100% vs. 30 or 24%: *p* < 0.005 for both comparisons) and over the left hemisphere for faces (*post hoc* tests for 100% vs. 30 or 24%: *p* < 0.01 for both comparisons) but remained stable across phase coherence levels for cars over the left and for faces over the right hemisphere (**Figure 4A**). Facial aging is mainly reflected in changes of skin textures and in altered tissue elasticity. As these changes can increase the amount of higher spatial frequency information only in the case of older face stimuli such low-level differences might explain the different phase-noise dependency of P1 for faces and cars. However, since age decisions for faces are mainly based on these factors (e.g., George and Hole, 2000), we have not equated the spectral content of the images. However, to test whether the significant hemisphere × category × coherence interaction is due to any differences in the spatial frequency content in the 100% phase coherent stimuli, we tested the effect of wrinkling/skin texture changes on the range of higher spatial frequency information. We plotted the spectral content of the 100% phase coherent stimuli by using the *sfplot* method of the SHINE toolbox (Willenbockel et al., 2010) and compared these functions for faces and cars at every morph level. Due to the small sample size, we used non-parametric ranked *t*-tests (point-by-point two-tailed Mann–Whittney *U* tests with Bonferroni-corrected *p* values). Although we found that the older the face stimuli, the more pronounced the spectral difference in the range of higher spatial frequency information when compared with car stimuli, it is worth noting that the spectral content of the youngest stimuli did not differ between the two categories. Next, we investigated the hemisphere × category × coherence × age interaction. The results suggest that the age information of the stimuli do not modulate the strength of the hemisphere-specific category effect reflected in the P1 component [hemisphere × category × coherence × age interaction: *F*(4,48) = 0.33, *p* = 0.86, η^2^ = 0.03, n.s.], arguing against the role of low-level spectral differences in explaining the results. Moreover, the age of the stimuli as a categorical factor neither had a main effect [*F*(2,24) = 2.78, *p* = 0.09, η^2^ = 0.19] nor had any significant two-way (any *p*s > 0.13), three-way (any *p*s > 0.25), or four-way interactions (any *p*s > 0.75) with other factors. Taken together with the fact that no significant differences in spectral content were observed between the youngest 100% phase coherent face and car stimuli, our results suggest that the observed hemisphere × category × coherence three-way interaction is not due to the low-level spectral differences in the original stimuli.

The latency of the P1 was significantly longer for cars when compared to faces [main effect of category: *F*(1,12) = 22.65, *p* = 0.0005, η^2^ = 0.65]. Adding phase noise to the stimuli increased the latencies of P1 component [main effect of coherence: *F*(1.27,15.27) = 9.7, *p* = 0.0008, η^2^ = 0.4468, *post hoc* LSD: 100% vs. 30 and 24%: *p* < 0.002 for both comparison]. This difference in latency was, however, similar for both categories [category × coherence interaction: *F*(1.71,20.5) = 1.77, *p* = 0.19, η^2 ^= 0.13; **Figure 4D**].

#### N170

We found a significant main effect of coherence for the amplitude of the N170 component, [*F*(1.48,17.78) = 71.45, *p* < 0.0001, η^2 ^= 0.86] reflecting the reduction of the N170 amplitude as the phase coherence decreases. It is worth noting that this effect was larger for the right when compared with the left hemisphere as suggested by the significant hemisphere × coherence interaction [*F*(1.28,15.38) = 5.52, *p* = 0.01, η^2^ = 0.32, **Figure 4B**]. Interestingly, N170 amplitudes did not show the typically observed face-specificity (see Kloth et al., 2013 for similar results): the N170 was almost identical for both faces and cars [main effect of category: *F*(1,12) = 0.49, *p* = 0.5, η^2^ = 0.04]. However, adding phase noise changed the category selectivity of the N170 as suggested by the significant category × coherence interaction [*F*(1.57,18.88) = 3.94, *p* = 0.03, η^2^ = 0.25].

As for the N170 latency, a strong tendency of category dependence was found, suggesting that face stimuli evoked an N170 component earlier than cars [main effect of category: *F*(1,12) = 4.32, *p* = 0.06, η^2 ^= 0.26]. The N170 was delayed by adding noise to the stimulus [main effect of coherence: *F*(1.06,12.77) = 7.82, *p* = 0.0024, η^2^ = 0.39, *post hoc* LSD: 100 vs. 30 and 24%: *p* = 0.005 for both comparisons]. In addition, a hemispheric asymmetry was also found in the noise-induced modulation of the N170 latencies [interaction between hemisphere and coherence: *F*(1.38,16.59) = 4.8, *p* = 0.0018, η^2^ = 0.29], which was due to shorter latencies for noise absent stimuli over the RH (LSD: *p* < 0.01), but similar latencies of the RH and LH for the other two noise conditions (LSD: *p*s > 0.34; **Figure 4E**).

#### P2

Supporting prior results (Philiastides et al., 2006; Nagy et al., 2009; Bankó et al., 2011), phase noise enhanced the amplitude of the P2 gradually [main effect of coherence: *F*(1.05,12.6) = 25.06, *p* < 0.0001, η^2 ^= 0.68]. Moreover, significantly larger P2 amplitudes were observed for face stimuli when compared to cars [main effect of category: *F*(1,12) = 40.17 *p* < 0.0001, η^2 ^= 0.77, **Figure 4C**]. This effect was more pronounced in the right hemisphere, as suggested by the significant interaction between hemisphere and category [*F*(1,12) = 6.17, *p* = 0.03, η^2 ^= 0.34]. It is worth noting, however, that the category selectivity of the component was not altered by the amount of altered phase coherency [interaction between category and coherence: *F*(1.25,15.02) = 1.6, *p* = 0.22, η^2 ^= 0.12]. Finally, the P2 component also showed a strong tendency toward a RH dominance [main effect of hemisphere: *F*(1,12) = 4.36, *p* = 0.059, η^2 ^= 0.27]. No significant effects and interactions were observed on the P2 latency values (**Figure 4F**).

#### The effect of stimulus ambiguity

**FIGURE 5:**
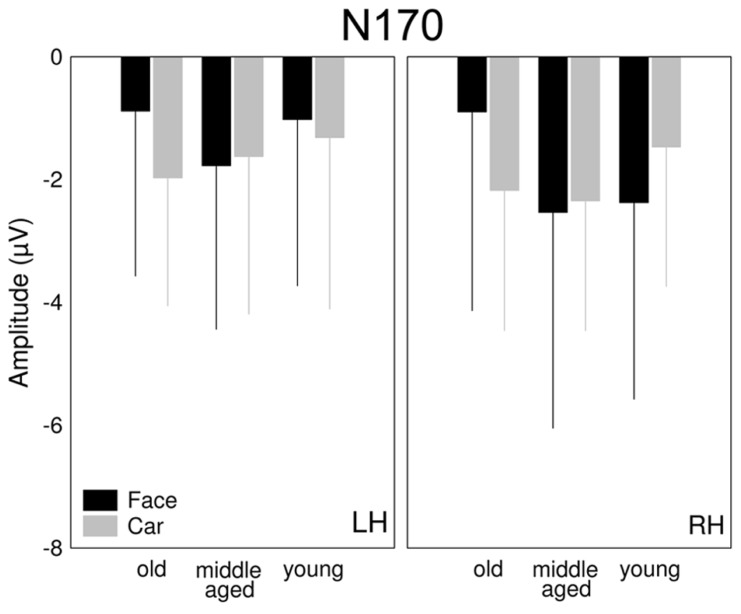
**The effect of stimulus ambiguity reflected on the N170 component.** Mean (±SD) of the amplitudes of the N170 for faces (black columns) and cars (gray columns) at different level of age (old vs. middle-aged vs. young).

Recent results suggest that stimulus ambiguity plays a role in determining the susceptibility of the N170 to stimulus adaptation (Walther et al., 2013). In order to test the effect of stimulus ambiguity and its noise dependence, we compared the early ERP components for the endpoints of morph continua (oldest and youngest stimuli) and for the most ambiguous (i.e., middle-aged) stimulus groups (see Materials and Methods). The first ERP component reflecting stimulus ambiguity was the N170: its amplitude was larger for middle-aged stimuli, as suggested by the main effect of age [*F*(1.9,12.75) = 10.13, *p* = 0.0006, η^2 ^= 0.46; *post hoc* LSD tests: old vs. middle-aged: *p* = 0.0004, young vs. middle-aged: *p* = 0.001 but young vs. old *p* = 0.66, respectively]. N170 also had a RH dominance for middle-aged and young stimuli but not for old ones (significant hemisphere × age interaction: *F*(1.5,18.1) = 12.53, *p* = 0.0002, η^2^ = 0.51, Fisher’s LSD tests: *p* < 0.0001 for both young and middle-aged stimuli and *p* = 0.31 for old stimuli, respectively). Interestingly, larger N170 amplitudes were measured for old cars when compared to faces, as suggested by the significant interaction between category and age [*F*(1.84,22.14) = 16.98, *p* < 0.0001, η^2 ^= 0.59; Fisher’s LSD: *p* < 0.0001 for old stimuli but not for middle-aged or young stimuli: *p*s > 0.13]. This effect was more pronounced in the RH [three-way interaction among category, hemisphere and age: *F*(1.65,19.78) = 8.13, *p* = 0.002, η^2^ = 0.4; **Figure 5**]. Another interesting result is that for noisy stimuli, the younger the faces were, the more pronounced the category effect was, as suggested by the significant category × coherence × age interaction [*F*(2.35,28.23) = 10.12, *p* < 0.0001, η^2^ = 0.46].

We observed larger P2 components for face than for car stimuli at every level of stimulus ambiguity, but this effect was the most pronounced for the old stimuli and weaker for the young ones (significant category × age interaction: *F*(1.52,18.2) = 8.01, *p* = 0.002, η^2^ = 0.4). No other effect of stimulus ambiguity was found.

## DISCUSSION

The goal of the present study was to test whether adding phase noise to a stimulus affects the neural processing of complex object stimuli in a category-specific manner by recording ERPs for faces and cars at different levels of phase coherences. Several previous ERP studies applied different types of noise to manipulate the difficulty of decisions about faces (Bentin et al., 1996; McKone et al., 2001; Jemel et al., 2003; Wild and Busey, 2004; Rousselet et al., 2008b; Bankó et al., 2011). They found that adding noise to faces (or reducing their phase coherence) affects the P1 – N170 – P2 ERP complex. In the case of the face-specific N170, it was found that phase noise reduces its amplitude dramatically (Jemel et al., 2003; Nagy et al., 2009; Bankó et al., 2011) and also prolongs its latency. In addition to the changes observed in the N170 amplitude, different types of noise manipulations made the behavioral task more difficult *per se* and this difficulty was linked to the P2 ERP component (Philiastides et al., 2006; Heekeren et al., 2008): the amplitude of this component was enhanced parallel to the difficulty of the task. Later, however, it was shown that the noise-induced modulation of the P2 reflects increased visual cortical processing demands instead of task difficulty *per se* (Bankó et al., 2011). Although the effect of phase noise on the electrophysiological correlates of face perception has been investigated extensively, the question whether noise-induced modulation of these components is specific to the category of faces has so far remained unanswered. The present results suggest that the early P1 component shows a category-dependent modulation of phase coherence.

The results of the electrophysiological recordings suggest that the first stage where category-dependent phase noise-induced modulation can be observed is the level of the early P1 component. In the noise-absent conditions, faces elicited larger P1 amplitudes when compared with cars in the RH, while no such category-specific effects were found in the LH (for similar results see Itier and Taylor, 2004). P1 is usually referred to as an early indicator of the endogenous processing of visual stimuli, and it is especially linked to spatial processing (Mangun, 1995). Recently, however, it has been shown that P1 reflects more than simply the low-level features such as contrast or luminance of the stimuli, it also indexes an early stage of visual processing, being sensitive to stimulus category such as faces (Taylor, 2002). As noted by Itier and Taylor (2002), P1 could reflect the holistic processing of a face as a face, whereas the later N170 component would reflect facial configurations. Adding noise to a face causes enhanced P1 in some studies (Schneider et al., 2007; Rousselet et al., 2008b; Nagy et al., 2009; Bankó et al., 2011, 2013), while others suggest that P1 is unaffected by such changes (Jemel et al., 2003; Wild and Busey, 2004; Horovitz et al., 2004). Interestingly, in both cases, it has been suggested that P1 is not involved in any aspect of face-processing, but it is rather involved in the sensory analyses of the images, irrespective of their content (Jemel et al., 2003). In the present study, the noise-induced modulation of the P1 showed category-sensitivity in a hemisphere-specific manner. Adding noise enhanced P1 amplitudes for cars over the RH but it had no effect over the LH, and vice versa; enhanced P1 values were observed for faces in the LH but not in the RH. These results suggest that the category-specificity of the noise-induced modulation of the ERP appear very early, that is, already at 100 ms after stimulus onset. Although we have equated all stimuli in luminance and matched their histograms, we did not equate the spectral content of faces and cars since larger amount of higher spatial frequency information is caused by wrinkling and reduced skin elasticity in the case of face stimuli. Since facial age decision is mainly based on this information (e.g., George and Hole, 2000) we did not equate the spectral content of images. This, however, raises the possibility that the category-specificity of the noise-induced modulation of the early P1 component is merely the effect of the different spectral content of the original stimuli. Indeed, several studies indicate that there are differences in sensitivity to the specific spatial frequencies both between different visual areas and between the two hemispheres (Ivry and Robertson, 1998). Our results, however, show that the category-specificity of the noise-induced modulation of the P1 is unaffected by the perceived age of the stimuli. Therefore, the amount of wrinkling that leads higher spatial frequency content in case of older faces does not modulate the results. In terms of hemispheric differences in sensitivity to specific spectral content, Sergent (1982) argued that the left hemisphere is more adept in processing high-frequency information, whereas the right hemisphere is more efficient in processing low-frequency information. This differential frequency processing account was supported by studies using tasks such as spatial frequency discrimination (Proverbio et al., 2002) and identification (Kitterle et al., 1990) or face recognition (Keenan et al., 1989). These results, however, would predict that 100% phase coherent faces with a larger amount of higher spatial frequency content would enhance the amplitude of the P1 component over the left hemisphere and adding phase noise would not affect P1 over the right hemisphere. Vice versa, 100% phase coherent cars with relatively lower spatial frequency content would enhance the P1 amplitude on the right hemisphere and adding phase noise would not affect this value when compared with the left hemisphere. However, our results show the complete opposite effect, suggesting that low-level features are not able to explain the described category dependence of P1. It is worth noting, however, that in a recent study, Motoyoshi et al. (2007) drew attention to other image-statistics that are sensitive to asymmetries in dark and light and can also affect the low-level properties of an image. Although we cannot exclude the possibility that these properties affect our results, it is unlikely that low-level differences between cars and faces are responsible for the results regarding the hemispheric asymmetries of the category-specific phase-coherence dependence of the P1 in the current study. It is also well known that there are hemispheric asymmetries in the processing of local versus global information processing. A left hemisphere advantage for responses to local features and a right hemisphere dominance for responses to global features was found in most studies (Weismann and Woldorff, 2005; Flevaris et al., 2010; Hsiao et al., 2013). Several lines of evidence suggest (e.g., the face inversion effect, the Thatcher illusion, or the composite face effect) that faces are not perceived as collections of isolated parts, but rather as holistic configurations (Yin, 1969; Thompson, 1980; Young et al., 1987). Most of the electrophysiological research studying the N170 emphasizes the specificity of the component to the structural encoding step of face processing (e.g., Bentin and Deouell, 2000; Eimer, 2000a,b). Other studies highlight the right hemisphere advantage of the component for manipulations of configural facial information, whereas the N170 in the left hemisphere is sensitive to the manipulations of featural facial information (Rossion et al., 1999; Scott and Nelson, 2006; Jacques and Rossion, 2007). This finding of different hemispheric specializations is consistent with evidence from neuroimaging studies. For example, in a PET study, Rossion et al. (2000b) have found hemispheric asymmetries for whole-based and part-based processing of faces in the fusiform gyrus in the sense that more pronounced right fusiform activation was observed for whole faces than face parts whereas this effect was reversed in the homologous left hemisphere brain region. fMRI studies have identified a number of areas – such as the fusiform face area (FFA; Kanwisher et al., 1997) and the occipital face area (OFA; Gauthier et al., 2000) in the extrastriate visual cortex – that respond more to pictures of faces than other objects, with a strong right hemisphere dominance (McCarthy et al., 1997; Haxby et al., 1999; Rossion et al., 2003). Presumably this right hemisphere dominance is reflected in the early P1 ERP component as well. Taken together with our findings on the P1 component, we can hypothesize that the activation of the right FFA is more robust to the amount of phase noise in the case of face stimuli. In other words, it suggests that while adding phase noise to faces alters rather featural but not configural information, the right hemisphere will be unaffected by this image manipulation. Although in a source localization study investigating the early stages of face processing, Herrmann et al. (2005b) have shown that the first step of cortical face processing (~100 ms after stimulus presentation) is localized in the fusiform gyrus, further studies are need to clarify the sensitivity of the FFA to image manipulations such as the effect of phase noise.

The electrophysiological results of the current study confirmed the classical noise-induced effects reflected in the N170 and P2 components (Nagy et al., 2009; Bankó et al., 2011): the N170 amplitude decreased for higher levels of phase noise in a stepwise manner (Jemel et al., 2003). The gradual decrease of the N170 as the faces and cars became more and more noisy can be accounted for by the sensitivity of the component to the visibility of the stimuli embedded in different amounts of noise. It can also be due to increased attentional resources as the amount of added phase noise reduced the coherence of the stimuli. The fact that the observed significant three-way category × coherence × hemisphere interaction measured on the P1 lost its hemispheric asymmetry in the N170 time window is suggestive of the involvement of additional neural mechanisms. Schneider et al. (2007) have shown that noise affects the neural correlates of upright and inverted faces differently. Many studies suggest that inversion results in faces being processed by a piecemeal, feature-by-feature strategy (Rossion et al., 2000a; Barton et al., 2001), more similar to non-face objects (Haxby et al., 1999; Rossion et al., 2000a; Rosburg et al., 2010; Kloth et al., 2013). As complex, non-face object stimuli such as cars are also processed in a feature-based manner, the category × coherence interaction observed in the N170 component is rather due to the effect of stimulus configuration on processing levels. The fact that N170 was similar in amplitude for 100% phase coherent car and face images suggests that individual exemplars of objects that are visually similar to faces and have homogeneous feature configurations can elicit comparable N170 responses (for similar stimulus comparisons and results see Kloth et al., 2013). It is worth noting, however, that these results do not suggest that similar encoding takes place for cars and faces, even when they are characterized by a similar, face-like configuration (Kloth et al., 2013). On the other hand, our results also confirm the classical noise-induced effects on the later P2 component as well (Nagy et al., 2009; Bankó et al., 2011). More positive peaks were observed for faces when compared to cars, especially in the RH, and gradually increased P2 components were measured parallel to the amount of added noise. In previous studies, the noise induced effect reflected in the later P2 component could be explained by two factors – adding noise to the stimulus increases the visual cortical processing demands (Bankó et al., 2011, 2013), or it results in enhanced responses of the neural populations representing stimulus uncertainty (Bach and Dolan, 2012). Since no significant category × coherence interaction was observed on the P2 component the results of the current study could not exclude either explanation.

In summary, in this electrophysiological study we explicitly compared the noise-dependence of face and non-face stimuli and we have found that the neural processing of different high-level categories diverge at a very early stage of stimulus processing, starting in the P1 time window.

## AUTHOR CONTRIBUTIONS

Designed the experiment: Kornél Németh, Gyula Kovács, Márta Zimmer; data acquisition: Kornél Németh, Petra Kovács, Pál Vakli; data analyses: Kornél Németh, Petra Kovács, Pál Vakli, Gyula Kovács, Márta Zimmer; interpretation of the data: Gyula Kovács, Márta Zimmer; provided materials: Kornél Németh, Petra Kovács, Pál Vakli, Márta Zimmer; wrote the article: Gyula Kovács, Márta Zimmer; proofed/revised the article: Kornél Németh, Petra Kovács, Pál Vakli, Gyula Kovács, Márta Zimmer.

## Conflict of Interest Statement

The authors declare that the research was conducted in the absence of any commercial or financial relationships that could be construed as a potential conflict of interest.
